# Health status and health-related quality of life of municipal waste collection workers – a cross-sectional survey

**DOI:** 10.1186/s12995-015-0065-6

**Published:** 2015-07-08

**Authors:** Marcial Velasco Garrido, Cordula Bittner, Volker Harth, Alexandra Marita Preisser

**Affiliations:** Institute for Occupational Medicine and Maritime Medicine (ZfAM), University Medical Center Hamburg-Eppendorf, Seewartenstrasse 10, 20459 Hamburg, Germany

**Keywords:** Refuse collection, Waste collection, Back complaints, HRQoL, EQ-5D-5L, Cross-sectional study

## Abstract

**Background:**

Waste collection workers are exposed to several occupational stressors which may affect their quality of life. Our aim was to assess the health status and health-related quality of life (HRQoL) of municipal waste collection workers of a big German city.

**Methods:**

Cross-sectional study with a non-random sample of 65 (62 male, 3 female) workers of the Hamburg sanitation department, volunteering to participate in the study. We assessed the prevalence of reported health complaints and health problems. HRQoL was assessed with the self-administered EQ-5D-5L questionnaire and its visual analogue scale (VAS).

**Results:**

The most common health problems were musculoskeletal complaints (back pain reported by 67.2 %, other musculoskeletal complaints 15.4 %). Asthma or chronic obstructive pulmonary disease (COPD) was reported by 15.4 % of the workers. All participants reporting having a diagnosis of asthma or COPD had been or were active smokers. Our findings indicate an impaired HRQoL among the investigated occupational group. Regarding EQ-5D 68.3 % reported at least “slight” problems in one or more dimensions, and almost one third (31.7 %) reported “no problems” in any dimension. Problems were most frequently reported in the dimension “pain/discomfort” (64.1 % of the workers). The mean VAS value was 80.9 (13.2). The presence of back pain was associated with limitations in HRQoL (RR 3.1; 95 %-CI 1.5-6.1). The EQ5D VAS score was statistically significantly lower among waste collectors with back pain (77.9 SD 14.1) compared to those with no back complaints (88.0 SD 7.6, p < 0.01).

**Conclusions:**

Back complaints are common among municipal waste collectors and are associated with considerable impairments in their HRQoL. Interventions to enhance ergonomic work are needed in order to reduce back complaints and enhance HRQoL in this occupational group.

## Introduction

Municipal solid waste generation varies across countries in relation to income level and rate of urbanization, with the OECD countries accounting for 44 % of the 1.3 billion tonnes of waste generated yearly worldwide [1]. The duties of waste collectors usually include picking-up waste from its point of production, emptying refuse containers onto trucks, and delivering the waste to disposal and processing facilities. Despite the variety of collection methods, the job has, in general, been described as ‘characterized by frequent lifting, carrying, pushing, and pulling of heavy objects’ [2]. Besides the recurrent heavy physical activity, waste collectors are also exposed to bacteria, endotoxins, mould, allergens, particulate matter, irritant inhalants, vehicle exhaust, atmospheric conditions, noise, and psychomental stress. These multiple work demands and hazards result in a higher incidence of health problems and injuries compared to other occupations [2–4]. As one would expect, there is growing evidence that the incidence of respiratory problems and musculoskeletal complaints is higher among waste collectors than in other occupations [5, 6].

Health-related quality of life (HRQoL) is a well-established aspect of health and general well-being, which can be measured with a variety of validated instruments [7]. In recent years, the issue of quality of life is increasingly being considered in occupational health research, with assessments of the relationship between occupational diseases and quality of life [8, 9]; and in relation to specific occupations [10–12].

The aim of our study was to assess (i) the prevalence of health problems and (ii) the HRQoL of waste collectors, and (iii) to investigate their associations.

## Methods

### Study design and participants

We conducted a descriptive cross-sectional study among workers of the Hamburg sanitation department. The study was part of a broader occupational and ergonomic field study which assessed the working conditions and job strain of municipal waste collectors with qualitative interviews, real-life observation and objective strain measurements (e.g. mobile spiroergometry) [13]. For the study, waste collectors were recruited by the human resources department of the municipal waste collection services of the City of Hamburg with the involvement of the safety engineer and collection tour planners. Participation in the field study was voluntary and rewarded with two days off. The sampling method was predefined by the ergonomic study and aimed at representing the broad spectrum of working conditions in the collection tours (i.e. areas with low and high population density, areas with frequent architectonic barriers, predominant type and size of container, etc.) and the two main work groups (street waste collectors and household waste collectors). The sample size was also determined by the ergonomic field study. The final study group consisted of 65 persons (62 male and 3 female) who were all active (i.e. not on sick leave) at the time of data collection (between January and August 2013). The majority of participants (71 %) were working in the household waste collection service.

### Measurements

Participants were invited to our Outpatient Clinic for Occupational and Environmental Health for a health check including a general clinical examination, 12-channel resting-ECG, lung function testing (spirometry and body plethysmography), as well as spiroergometry as described elsewhere [14]. All participants signed a written informed consent document after the examining physician had explained the health check-up and the study. Before undergoing the clinical interview and clinical examinations, the participants were asked to answer a questionnaire regarding current and past health problems and to self-assess their HRQoL. The health problems questionnaire listed organ systems and asked (yes/no questions) whether the participant ‘suffered or had suffered from any disease of that system, providing examples of the most common conditions for each system. It also included questions on smoking habits and medical treatments. The questionnaire was used to support the subsequent clinical interview.

HRQoL was self-assessed with the EQ-5D-5L instrument [15, 16] in its German version. The instrument consists of a descriptive system with five dimensions (‘mobility’, ‘self-care’, ‘usual activities’, ‘pain/discomfort’, ‘anxiety/depression’) and a visual analogue scale (VAS). In the descriptive system, the participant is asked to report his/her health state for each dimension on the present day (i.e. the day of answering the questionnaire) by ticking the most appropriate answer among five levels (‘no problems’, ‘slight problems’, ‘moderate problems’, ‘severe problems’, and ‘extreme problems’; the word ‘problems’ is replaced by “pain or discomfort’ in the fourth dimension and by ‘anxious or depressed’ in the fifth dimension). For each dimension, the answers are coded with a 1-digit number from ‘1’ (‘no problems’) to ‘5’ (‘extreme problems’). The digits of the 5 dimensions are combined into a 5-digit number which describes the overall health state of the respondent [17]. For example ‘11111’ indicates no problems in any of the dimensions; ‘21131’ would indicate slight problems in mobility, moderate pain and discomfort and no problems in the other dimensions. In the EQ VAS, the self-rated health is visualized on a 20 cm vertical, visual analogue scale with endpoints labelled ‘the best health you can imagine’ (‘100’) and ‘the worst health you can imagine’ (‘0’) and to write the number marked in the scale on a box [17]. The time horizon for the VAS is also the present day.

The clinical interview was structured around the above-mentioned health problems questionnaire and aimed at ensuring the collection of relevant data on health problems.

### Statistical analysis

Descriptive statistics are presented as median (interquartile range, IQR) or mean (standard deviation, SD) for continuous variables, and as frequency and percentage for categorical variables. The associations between health problems commonly reported and HRQoL were analysed calculating relative risk (RR) with 2x2 contingency tables and Fisher’s exact test. Mean differences across groups were tested with unpaired Student’s t. For multivariate analysis, logistic regression was performed adjusting for age, BMI and current smoking status, since these have been associated before with impairments in quality of life among adults [18, 19]. Two-tailed P values were calculated. Statistical analyses were carried out using PASW Statistics Software 18.0 (SPSS Inc., Chicago, IL, USA, IQR and Epi Info™ 7.1.3.3 (Centers for Disease Control and Prevention, Atlanta, GA, USA).

The study was approved by the Ethics Committee of the Hamburg Medical Association (register no. PV4524).

## Results

Table 1 reports the characteristics of the study participants in comparison with the source population. Despite slight differences, the self-selected non-random sample was similar to the source population regarding age, gender distribution and years on the job. Vocational training prior to employment in the municipal waste collection services had been done more frequently by study participants. In general, waste collectors represent an aged collective, with the median age at 47.5 (IQR 10.6) years and 66.1 % of participants being above 45 years old.

**Table 1:** Participant characteristics compared to source collective

	Study participants				Source collective			
	(N = 65)				(N = 1,544)			
	Mean	SD	Number	Percent	Mean	SD	Number	Percent
Age (years)	45.5	8.3			46.5	9.4		
Gender								
Female			3	4.6			31	2.1
Male			62	95.4			1513	97.9
Previous vocational training								
Yes			52	80			1109	71.8
No			13	20			435	28.2
Years working for the municipal waste collection service	20.4	9.1			18.6	9.9		

Mean body mass index (BMI) was 28.3 kg/m^2^ (SD 3.8). Overweight (BMI ≥25 kg/m^2^) was highly prevalent among participants (83.1 %); obesity (BMI ≥30 kg/m^2^) was present in 29.2 % of the waste collectors. The prevalence of smoking among the participants was 35.4 %; only 22.4 % reported to have never smoked.

Table 2 summarizes the results regarding health problems. A total of 89.2 % of the participants reported having at least one health problem. Considering vision limitations as a health problem raised the prevalence of health problems to 93.8 %. About one third reported currently being under medical treatment (29.2 %) or on long-term drug treatment (33.8 %). Most of the drug therapies (60 %) targeted cardiovascular diseases and cardiovascular risk factors. 11 % of the participants reported that they took antidepressant drugs.

**Table 2:** Reported diseases and health problems. [N = 65, multiple answers possible]

	Number	Percent
Back complaints	43	67.2
Vision problems or eye diseases	38	58.5
Long-term drug treatment	22	33.8
Allergies	21	32.3
Currently under medical treatment	19	29.2
Cardiovascular diseases	15	23.1
Respiratory diseases	13	20.0
Hearing problems or ear diseases	12	18.5
Gastrointestinal diseases	11	16.9
Other musculoskeletal complaints	10	15.4
Psychiatric problems	10	15.4
Dermatological problems	10	15.4
Vertigo, Syncope	8	12.3
Neurological diseases	5	7.7
Diabetes	3	4.6

The most common health problem was back pain, reported by 67.2 % of the participants. In the clinical examination, spinal percussion and/or palpation were painful in 11.5 % of the waste collectors and the finger-floor distance was over 10 cm in 19.3 % of the study group. Other musculoskeletal complaints were reported by 15.4 %.

According to the answers to the questionnaire, asthma and COPD had a prevalence of 7.7 % each. All participants reporting a diagnosis of asthma or COPD had been or were active smokers. Lung function tests yielded an obstructive pattern (FEV_1_/FVC <70 %) in 21.5 % of the participants. Among those reporting a diagnosis of respiratory disease, 38.4 % showed an obstructive pattern (3 of them had reported a diagnosis of asthma and 2 of COPD).

All but one participant answered the EQ-5D questionnaire; one participant did not rate the “anxiety/depression” dimension. Almost one third (31.7 %) of the participant waste collectors rated their health status as ‘without problems’ in any 5 dimensions (i.e. 11111) (Table 3). The rest reported at least ‘slight’ problems in at least one dimension. The dimension for which problems were most frequently reported was ‘pain/discomfort’ with 46.9 %, and 17.2 % reported ‘slight’ or ‘moderate’ problems respectively. Almost one third (28.1 %) reported ‘slight’ or ‘moderate’ problems in the dimension ‘mobility’. The only dimension for which ‘severe’ problems were reported was ‘anxiety/depression’. None of the participants reported any problems in the dimension ‘self-care’.

**Table 3:** Frequency of health status (i.e. response patterns to the five-dimension descriptive part of EQ5D). [N = 63, frequency shown for patterns reported by more than one participant]

EQ5D Health Status	Number	Percent
11111	20	31.7
11121	14	22.2
21121	6	9.5
11122	4	6.3
11112	3	4.8
31231	3	4.8
11222	2	3.2
21122	2	3.2
21131	2	3.2

**Fig. 1:**
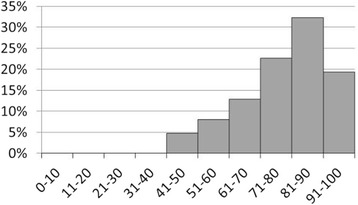
Distribution of VAS-scores. [N = 62; 3 missing values]

The distribution of the VAS scores is shown in Fig. 1. All but 3 participants rated their HRQoL in the VAS. The mean EQ5D VAS score was 80.9 (SD 13.2); the lowest score was 50, the highest 100.

We explored the association between reported health problems and HRQoL in contingency tables for the conditions reported by at least one fifth of the participants (Table 4). Reported back complaints were associated with lower quality of life in the five-dimensional part of the EQ5D. The majority (87.8 %) of waste collectors with back pain had an HRQoL other than 11111. All participants reporting problems in the dimension ‘mobility’ had also reported back pain. The majority (88.4 %) of participants reporting back pain had also reported at least ‘slight’ problems in the dimension ‘pain/discomfort’. Back complaints were also associated with lower VAS score. The VAS score among waste collectors without back pain was statistically significantly higher than the VAS score of participants with back pain (88.0 SD 7.6 vs. 77.9 SD 14.1, p = 0.0051). VAS score was also statistically significantly lower among waste collectors under current medical treatment compared to those not in treatment (75.8 SD 14.8 vs. 85.0 SD 10.1, p = 0.0001) and among waste collectors reporting allergies compared to those with no allergy (75.9 SD 15.1 vs. 83.3 SD 11.7, p = 0.0408). For all other conditions listed in Table 4, there were no statistically significant differences in mean VAS score, although mean VAS score was always lower among waste collectors reporting the health problem or disease.

**Table 4:** Health problems and health-related quality of life limitations.

Health problem	EQ5D 11111 (no problems)		EQ5D other than 11111 (at least slight problems in at least one dimension)		RR (raw)	95 % CI
	Number	Percent	Number	Percent		
Back complaints	5	25.0	36	85.7	3.07	1.54-6.10
Vision problems or eye diseases	11	55.0	26	60.5	1.01	0.76-1.52
Long-term drug treatment	6	30.0	16	37.2	1.11	0.79-1.55
Allergies	7	35.0	14	32.7	0.97	0.67-1.39
Currently under medical treatment	5	25.0	14	32.6	1.12	0.79-1.57
Cardiovascular diseases	3	15.0	12	27.9	1.24	0.89-1.72
Respiratory diseases	2	10.0	11	25.6	1.32	0.97-1.80
RR: relative risk						

The association between back complaints and any limitation in quality of life (i.e. health status other than ‘11111’) was still statistically significant after adjusting for age, BMI and current smoking status with logistic regression (OR 14.1; 95 %-CI 3.4-59.0).

## Discussion

This is the first study to examine health status and quality of life using a validated instrument, the EQ-5D, of waste collection workers, although sample size was small. Interestingly, we observed a high prevalence of back complaints which was strongly associated with limitations in health related quality of life in this occupational group.

Previous studies have reported a higher prevalence of respiratory problems, including workplace-related respiratory complaints, among waste collectors than in the general population [3, 5, 6, 20]. This might be explained through the exposure against allergens and toxins contained in waste, seasonal allergens, and infectious agents [2]. Despite a higher prevalence of smokers and ex-smokers as in the German general population [21], we unexpectedly found a similar prevalence of asthma and COPD among our sample of waste collection workers as reported by the German general population [22]. However, on the basis of lung function testing, we identified 9 subjects with obstructive ventilatory abnormalities who had not reported having a respiratory disease, indicating the possibility of a higher total prevalence of respiratory problems in our sample, as one would expect. A limitation of our study is that we did not ask about workplace-related respiratory complaints. This could explain the discrepancy between previous papers and ours regarding respiratory diseases.

In the present study we found a higher prevalence of back complaints among waste collectors compared to the German general population [23]. This result is in accordance with previous reports in the literature [2, 6]. Although in our study we could not establish whether the reported back complaints were workplace-related, the available evidence indicates that waste collectors may have a higher incidence of musculoskeletal complaints than other occupations, equivalent to the findings of Kujier et al. [6]. In addition, musculoskeletal disorders have been reported as accounting for about one fifth of sickness absence in the waste industry [24]. This is not surprising since handling waste containers represents a considerable strain for the musculoskeletal system, particularly of the back, through the combination of trunk lateral flexion, bending and torsion, as shown in ergonomic observations [25]. The waste collectors included in our study pick up two-wheeled waste containers (120 L volume) from houses and cellars (partially overcoming stairs) and also shift large four-wheeled waste containers (240 L) from storerooms to the truck. The ergonomic field study demonstrated through mobile spiroergometry (measurement of oxygen uptake) a high physical demand in the job of both refuse collectors and street cleaners [14]. The qualitative interviews also showed that the job is psychologically demanding because of time pressure, accident risks and perceived unfairness of quality control [26], which can also represent a strain related to musculoskeletal complaints. Further, we found a high prevalence of overweight in our sample. Overweight has been associated with back pain and other musculoskeletal complaints [27, 28] and may represent an additional strain for the musculoskeletal system.

Our findings indicate an impaired HRQoL among this occupational group as compared to the reference population: Whereas 47.5 % of a representative sample of the German general population (i.e. including unemployed, chronically ill, etc.) reported the ideal EQ-5D health status [29], only one third (31.7 %) of the waste collectors rated their health status as without problems in all of the 5 dimensions. Taking the VAS score, HRQoL of waste collectors is comparable to that of German population samples. In a survey in the years 2002/2003, VAS score among employees was 82.3 (SD not reported), whereas the general population had a mean VAS score of 77.4 (SD 19.0) [30]. A more recent survey (data from 2006-2008) reported a mean VAS score for the general German population of 79.8 (SD not reported) [31]. However, comparisons with published results for the general population are to be interpreted carefully since weighting for sociodemographic factors is not possible.

We found an association between back complaints and limitations in the quality of life of municipal waste collectors. After adjusting for BMI and smoking back pain remained the most important factor limiting HRQoL, although the precision of our estimate was limited by the small size of our study (95 % CI 3.4-59.0). Back pain has been described before as a limiting factor of HRQoL among industrial workers [32] and also among sedentary office workers [33]. Although our cross-sectional study design does not allow drawing conclusions on causality, the frequent limitations in the dimensions ‘pain/discomfort’ and ‘mobility’ of the EQ-5D point at back complaints as being one of the explaining factors for the limitation of overall HRQoL among the examined group of municipal waste collectors. This is also supported by the differences in VAS scores according to problems in the dimensions ‘pain/discomfort’ and ‘mobility’ of the EQ-5D. The mean VAS score among those without problems in the dimension ‘pain/discomfort’ indicated better health than among waste collectors with problems in this dimension (86.9 SD 7.4 vs. 77.3 SD 14.1, p = 0.0077). Similarly, the VAS score showed better results among those without problems in the dimension ‘mobility’ (83.2 SD 11.4 vs. 74.7 SD 14.8, p = 0.026). All in all our study shows an association between back complaints and limitations in HRQoL. Interventions addressing ergonomic handling of waste containers and street sweeping may reduce back complaints and finally contribute to improve quality of life among these workers.

To our knowledge, only few studies have been conducted on HRQoL among waste collectors and these were limited to the special group of gatherers of recyclable materials in Brazil. One study used the WHOQOL-100 instrument but reported results too briefly to draw any sound conclusions [34], another one did not report having used a validated instrument to assess HRQoL and actually was focused on personal satisfaction [35], thus not being comparable with our work.

To our knowledge ours is the first published systematic assessment of HRQoL among a group of municipal waste collectors using a validated instrument, the EQ-5D.

Besides the cross-sectional design, which lacks the temporary dimension for cause-effect relationships, another limitation of our study is the small self-selected non-random sample. Our group included workers from the household collection (including organic waste collection) and the street sweeping services. According to age, gender distribution and length of work in waste collection, the participants can be considered to be representative of the employees of the municipal waste collection services.

Recruitment of the participants by the human resources department could have introduced selection bias, i.e. through recruitment of healthier workers. Indeed all participants were active at the time of participating in the study – i. e. not on sick leave – thus a healthy worker effect could have led to underestimation of the prevalence of disease. Potential participants were approached in order to represent the broad spectrum of working conditions in the collection department and participated voluntarily. Since absolute confidentiality was warranted in our study and the health check and interview were conducted in our Institute, we do not think that reporting of symptoms and health problems has been relevantly affected by the recruitment method. Being afflicted with health problems could have been the motivation to participate in the volunteer study, which would bias our results. Since our study was only a part of a broader ergonomic study in which field observations had the prominent role, we do not think the main motive to participate was own health complaints. In the qualitative interviews of the broader ergonomic study, the reported main motivations to take part in the study were to show how hard the job of a waste collector is and to contribute to improvements in the company [26].

## Conclusions

In summary, we found back complaints to be a frequent health problem among municipal waste collection workers, more frequent than in the general population. These complaints are associated with limitations in health-related quality of life and probably explain at least some of these impairments. Interventions to enhance ergonomic work processes in this occupational field are warranted in order to reduce musculoskeletal problems and should be evaluated taking HRQoL into account.

## Abbreviations

BMI: Body mass index
CI: Confidence interval
COPD: Chronic obstructive pulmonary disease
HRQoL: Health related quality of life
IQR: Interquartile range
OR: Odds ratio
RR: Relative risk
SD: Standard deviation
VAS: Visual analogue scale
